# UK clinical practice guidelines for the management of patients with constitutional POT1 pathogenic variants

**DOI:** 10.1136/jmg-2025-110638

**Published:** 2025-05-11

**Authors:** Olga Tsoulaki, D Gareth Evans, Khushboo Sinha, Neil Rajan, Farah Bakr, Helen Hatcher, Andrea Napolitano, Elena Finn, Sunil Iyengar, Aslam Sohaib, Timothy J Sadler, Claire Forde, Emma Roisin Woodward, Terri P McVeigh, Marc Tischkowitz, Fiona Lalloo, Helen Hanson

**Affiliations:** 1 St George’s University of London, London, UK; 2 Manchester Centre for Genomic Medicine, Manchester University NHS Foundation Trust, Manchester, UK; 3 Manchester Centre for Genomic Medicine, Division of Evolution, Infection and Genomic sciences, The University of Manchester, Manchester, UK; 4 St John’s Institute of Dermatology, Guy's and St Thomas’ Hospitals NHS Trust, London, UK; 5 Translational and Clinical Research Institute, Newcastle University, Newcastle upon Tyne, Tyne and Wear, UK; 6 Department of Dermatology, NIHR Newcastle Biomedical Research Centre, Newcastle upon Tyne, Newcastle upon Tyne, UK; 7 Addenbrooke’s Hospital, Cambridge University Hospitals NHS Foundation Trust, Cambridge, UK; 8 Sarcoma Unit, The Royal Marsden NHS Foundation Trust, London, UK; 9 Clinical Studies, The Institute of Cancer Research, London, UK; 10 Early Diagnosis and Detection Centre, Royal Marsden Hospital NHS Trust, London, England, UK; 11 Department of Haematology, The Royal Marsden NHS Foundation Trust, London, UK; 12 Department of Radiology, Royal Marsden Hospital NHS Trust, London, UK; 13 Department of Radiology, Cambridge University Hospitals NHS Foundation Trust, Cambridge, UK; 14 Cancer Genetics Unit, Royal Marsden Hospital NHS Trust, London, UK; 15 Department of Medical Genetics, NIHR Cambridge Biomedical Research Centre, Cambridge, Cambridgeshire, UK; 16 Royal Devon University Healthcare NHS Foundation Trust, Exeter, UK; 17 Department of Clinical and Biomedical Sciences, University of Exeter Medical School, Exeter, UK

**Keywords:** Germ-Line Mutation, Genetic Predisposition to Disease

## Abstract

Constitutional or germline pathogenic variants (GPVs) in *protection of telomeres 1 (POT1*) are associated with a variety of tumours resulting in the recognition of POT1-tumour predisposition syndrome (POT1-TPDS). These tumours may include cutaneous melanoma, angiosarcoma, haematological malignancy and brain tumours. Due to the rarity of *POT1* GPVs and limited available data, the overall lifetime cancer risks for individuals with POT1-TPDS are unclear. Furthermore, there is scant evidence to support the role of surveillance in early cancer detection in this patient group. A recent international publication suggested a surveillance protocol similar to that used in Li-Fraumeni Syndrome (LFS) could be offered to *POT1* pathogenic variant carriers, particularly where there are LFS-like features. However, current evidence for POT1-TPDS is not supportive of an equivalent lifetime cancer risk. Given the inclusion of *POT1* in the National Test Directory in England and the need for UK-based guidance, an expert group undertook a literature review to assess the phenotypic spectrum of POT1-TPDS and to provide lifetime risk estimates of *POT1*-associated cancers. The available evidence was shared with a small working group of experts that included clinical geneticists, dermatologists, sarcoma specialists, haematologists and radiologists to cover all aspects of the cancers most commonly associated with POT1-TPDS. Following structured expert group discussions, we achieved consensus on best practice recommendations for a POT1-TPDS UK management protocol.

## Background

 A growing body of epidemiological evidence has shown that excessively long telomeres are associated with increased cancer predisposition. This finding led to the identification of genomic rare variants in telomeric shelterin components (reviewed in a study by Kim *et al*^1^). Protection of telomeres 1 (POT1) is a component of the shelterin complex that has a critical role in regulating telomere length.^1 2^ Germline pathogenic variants (GPVs), including likely pathogenic (LP) variants, in *POT1* have been associated with a variety of tumours resulting in the recognition of POT1-tumour predisposition syndrome (POT1-TPDS). Two independent studies reported an association of GPVs in *POT1* with familial melanoma.^3 4^ Further core ‘*POT1* associated tumours’ including angiosarcoma (AS), haematological malignancy and brain tumours have been suggested.^5^,^7^ A wide phenotypic spectrum of cancer types, including other sarcomas, colorectal and thyroid cancer, has been described within POT1-TPDS families.^8 9^

POT1-TPDS is inherited in an autosomal dominant manner. Identifying GPVs in *POT1* may help explain a personal or family history of cancer, help tailor early cancer detection and prevention strategies in individuals with a *POT1* GPV and facilitate predictive (also known as pre-symptomatic or familial) testing and onward management of at-risk relatives. With respect to genetic testing in clinical practice, in England, *POT1* has recently been included in the familial melanoma panel. It has also been added to the Li-Fraumeni Syndrome (LFS) gene panel.^10^ However, there is very limited systematic evidence demonstrating an association of POT1-TPDS with LFS or Li-Fraumeni-Like (LFL) syndrome.^6 11^ In other countries and in commercially available panels, *POT1* is often included as part of a wider cancer predisposition panel test.

Given the small number of individuals identified with *POT1* GPVs, the overall lifetime cancer risks for individuals with a GPV in *POT1* are unclear and data on long-term follow-up of identified families are limited. Current estimates of risk are likely to be overestimated, and more studies are needed to assess the role of *POT1* in cancer susceptibility. Furthermore, the review of *POT1* variants identified in some families based on the current American College of Medical Genetics and Genomics (ACMG) variant classification criteria has suggested some variants may not be clinically relevant.^9^ To date, no genotype-phenotype correlations have been confirmed.

At present, there are no peer-reviewed published national or international guidelines that make recommendations for cancer surveillance in individuals with *POT1* GPVs. A recent international publication suggested guidelines for *POT1* tumour surveillance relating to melanoma, AS, chronic lymphocytic leukaemia (CLL) and glioma.^8^ The authors suggested that due to the similarity of some cancers to LFS and LFL syndrome, it may be appropriate to consider similar surveillance to that used in LFS, including whole body MRI (WB-MRI), particularly where the family history has LFS type cancers. This guidance was also supported by a more recent publication describing the cancers identified in three large families with *POT1* LP variants, ascertained through clinical genetics on the basis of personal and family histories of cancer.^9^ However, there is no firm evidence to suggest that *POT1* heterozygotes have lifetime cancer risks comparable to *TP53* heterozygotes or that they would benefit from a similar approach to surveillance. Most of our current understanding about cancer phenotype and risk in POT1-TPDS has arisen from case reports where cases have been selected based on specific clinical characteristics and thus are likely to be subject to ascertainment bias.^6^ A recent review highlighted the need for a revised classification of *POT1* variants according to ACMG recommendations, as the assessment of pathogenicity of these variants was often incomplete. Without robust information on cancer risk and/or the utility of surveillance for *POT1* heterozygotes, a more cautious approach to surveillance may be required.^12^

Given the emerging phenotype of this recently described cancer predisposition syndrome and the possibility of increased identification of *POT1* GPVs via large gene panels, the clinical guideline group of the CanGene-CanVar project, in collaboration with representatives from the UK Cancer Genetics Group (UKCGG) and a small working group of experts in cancers associated with POT1-TPDS, held two virtual meetings with the aim to address three main areas:

1. Review current evidence regarding the phenotypic spectrum of POT1-TPDS.
2. Provide lifetime cancer risk estimates for individuals with *POT1* likely pathogenic/pathogenic (LP/P) variants, overall and for specific cancers.
3. Discuss best practice regarding surveillance of individuals with *POT1* LP/P variants, including type, frequency and age of commencement of surveillance, if indicated.

### Scope of guidance

The overarching aim of the guidance is to provide a resource to healthcare professionals to aid clinical management of individuals with a GPV in *POT1* by integrating available information from the literature, with respect to lifetime cancer risks, alongside opinions of an expert group and consistent with the surveillance offered for other cancer predisposition syndrome within the National Health Service/publicly funded healthcare. For the purpose of these guidelines, only phenotypes with a clear association with POT1-TPDS were considered. It is important to note that these guidelines should support clinical practice, but they should not replace clinical judgement.

## Methods

### Pre-meeting preparation

Prior to the expert group meetings, a comprehensive literature review through a search of PubMed and citation chasing was performed to identify key publications on cancer incidence and surveillance in POT1*-*TPDS to inform discussions. A recent article proposing surveillance recommendations for individuals with *POT1* tumour predisposition syndrome was selected as a key publication to create a series of questions to be addressed at the expert group meetings.^8^ A background document summarising current recommendations for *POT1* testing and surveillance was drafted. Lifetime cancer risk estimates for individuals with *POT1* GPVs were calculated based on the lifetime cancer risks in the UK population, the relative risks from case–control studies and using the gnomAD non-cancer data set (see results and table 1). The background document and lifetime cancer risk estimates were circulated to all attendees via email prior to the meeting. The aim of the pre-meeting reading materials was to ensure attendees could review the available evidence and issues to be discussed prior to the meeting, and to equip attendees with up-to-date information on which to inform their opinions on a proposed POT1-TPDS UK surveillance protocol.

**Table 1:** Lifetime cancer risk estimates in individuals with *POT1* GPVs

Cancer	Lifetime risk (UK)^39^	Relative risk (case–control studies)	Lifetime risk estimates for *POT1* heterozygotes†	References
Melanoma	1 in 36 males* 1 in 47 females*	OR=2.11 0.24% in non-melanoma and 15/2928 0.51% in melanoma 3/3720 sporadic melanoma	3.3–5% (1 in 20–30)	^3 4 14 39^
Angiosarcoma	1% of cancers are sarcomas; 1% angiosarcomas	3/30 sporadic tumours with angiosarcoma (10%)	2%	^6 7 39^
	0.005%	Possibly 1 in 200 000 will have POT1-associated angiosarcoma in their lifetime	1 in 2795 carry loss-of-function variant in gnomAD; risk~1–2%	
			May be higher with missense variants including NM_015450.3:c.349C>T	
CLL	0.5% (1 in 200)	OR=3.6	1–2%	^21 39^
		6/1083 cases (0.55%)		
Glioma	0.5% (1 in 200)		No good case–control data	^28 39^
Overall	1 in 2 (50% males, 45% females)	OR=2.3 non-cancer controls 0.03577%	~60%	^39^
		48/134,187 (many only had sequencing~1 17 000)		
		6/7269 (0.0825) cases in gnomAD		

* Risks reflect those for white Europeans not black and South Asians whose risks will be much lower.

† Calculated from attenuating the multiplication of lifetime risk and relative risk.

CLL, chronic lymphocytic leukaemia; GPVs, germline pathogenic variants; POT1, protection of telomeres 1.

### Meeting format

Experts in specific fields related to POT1-TPDS (n=18) were invited to attend the first consensus meeting on 12 July 2023. 15 experts attended this meeting which included clinical cancer geneticists (n=8), dermatologists (n=2), sarcoma specialists (n=3), a haematologist (n=1) and a radiologist (n=1). Further input from radiology was sought following the meeting. An introductory talk was delivered by a member of the organising committee to highlight the aims of the meeting and provide a context-specific summary to attendees. All experts were consulted for their opinions on surveillance recommendations and were asked about their current practice and experiences. The following topics were covered at the meeting:

- General comments on the background document:
  - Existing literature.
  - Cancer types associated with POT1*-*TPDS (cutaneous melanoma, AS, CLL and glioma).
  - Lifetime risks (overall and for specific cancers).
- Discussion on surveillance recommendations for each cancer type.

Experts in a specific cancer field (eg, melanoma, AS, CLL and glioma) led the discussion relating to surveillance recommendations based on the following key questions (online supplemental information):

1. What is the clinical utility of surveillance for each cancer type for individuals with *POT1* LP/P variants?
2. If there is clinical utility, should surveillance be offered to all individuals with *POT1* LP/P variants or only to those who have additional risk factors?
3. If there is not any established clinical utility of surveillance, should any surveillance be considered depending on the family history and/or judged on a case-by-case basis?
4. Should all individuals with *POT1* LP/P variants be offered advice about symptom awareness? If yes, at what frequency?

After each discussion, there was debate by all of the expert group until agreement was reached on proposed UK surveillance guidelines. The recommendations for CLL, glioma and AS were the consensus opinion of all experts. A follow-up dermatology-focused meeting was held on 6 September 2023 attended by a small group of clinical geneticists (n=3) and dermatologists (n=3) to refine the melanoma surveillance best practice statements made by the expert group.

Based on the agreed recommendations, a patient information leaflet was developed. Patient representatives from the CanGene-CanVar Patient Reference Panel were consulted for their opinion on the comprehensibility of the information leaflet.

### Meeting report

A meeting summary document was circulated to all stakeholders via email (n=18), including those who were unable to attend the initial meeting, to seek specific feedback on the document and their input into the proposed UK surveillance recommendations. The recommendations were presented at the Joint UK/Dutch Cancer Genetics Meeting in March 2024.

### Evidence grading

As is typical for many rare diseases, the volume of peer-reviewed evidence available to consider for these guidelines was small and came from a limited number of articles, which typically reported on small samples or series. To balance the weight of both published evidence and quantify the wealth of expert experience and knowledge, we have used a scale for evidence grading that was introduced in the European Reference Network (ERN) GENTURIS Cancer Surveillance Guideline for individuals with *PTEN* hamartoma tumour syndrome: (1) strong evidence: consistent evidence and new evidence unlikely to change recommendation and expert consensus; (2) moderate evidence: expert consensus or majority decision but with inconsistent evidence or significant new evidence expected and (3) weak evidence: inconsistent evidence and limited expert agreement.^13^

## Results

### Prevalence and penetrance

*POT1* has been proposed as a key cutaneous melanoma susceptibility gene identified in 2–4% of *CDKN2A/CDK4* negative families.^3 4 14^ Its role in predisposition to other cancers is not yet fully established. The malignancy risk associated with *POT1* GPVs is currently unclear due to limited data on long-term follow-up of individuals ascertained through cascade testing following a positive diagnostic test in an affected individual. A total of 49 *POT1* variants purportedly associated with cancers other than cutaneous melanoma were identified in a recent comprehensive literature review.^12^ The pathogenicity classification of these variants ranged from benign to pathogenic. The review highlighted the need for revised classification of these variants due to issues relating to the absence of segregation studies and biased data sets. Functional studies, other than telomere length assessment, are also lacking.^2^ Overall, current estimates of risk are likely to be overestimated due to ascertainment bias and more studies are needed to assess the role of *POT1* in susceptibility to cancer.

Estimates of lifetime cancer risks for individuals with *POT1* LP/P variants are demonstrated in table 1. These were generated by attenuating the multiplication of the lifetime cancer risks in the UK population published by Cancer Research UK by the relative risks from case–control studies and using the gnomAD non-cancer data set. This is necessary because relative risks vary with age. For example, for *BRCA1,* there is an overall relative risk of 10-fold for breast cancer. However, the relative risk aged 20–30 years is >30-fold but only 3-fold aged 70–79. To generate reliable lifetime risks it is necessary to have relative risks in each 10-year age cohort or have a large enough cohort.

Cutaneous melanoma is the most reported malignancy associated with POT1-TPDS. There is also recent evidence to suggest that POT1-associated melanomas are more likely to demonstrate spitzoid morphology, an uncommon histopathological finding typically seen in paediatric melanocytic tumours.^15 16^ While different studies have different definitions of familial melanoma, most included pedigrees of at least two cases of melanoma. In 2014, Robles *et al* described constitutional variants in *POT1* in almost 4% (4/105) of familial melanoma pedigrees.^4^ In the same year, Shi *et al* reported a missense *POT1* variant in 8.7% (4/56) of cutaneous melanoma families.^3^ More recently, *POT1* constitutional variants were identified in 1.75% (4/228) of familial melanoma pedigrees in a Spanish cohort.^17^ A founder *POT1* variant has been reported in individuals of Jewish ancestry and patients from the Romagna region in Italy,^5 18 19^ but the frequency in these populations compared with other populations is not well established, such that ethnicity is not a deciding factor in indications for testing. Interestingly, a recent cutaneous melanoma case–control cohort study demonstrated that the contribution of *POT1* to melanoma risk burden in the general population was only 0.5% with an OR of only 2.1 (95% CI 0.89 to 5.00).^14^ Multigene panel testing in a large cohort of patients assessed retrospectively demonstrated a clear association with melanoma (OR=7.03, 95% CI 4.7 to 10.5; p<0.001) and suggested loss of function as a disease mechanism.^20^ However, the latter paper is not a true case–control study, as the control group comprised individuals with negative multigene panel testing, rather than a population-based cohort. The sample only included 308 melanoma cases with the great majority of tests carried out for breast cancer (n=6313), which had a negative correlation with *POT1* (OR=0.71, 95% CI 0.53 to 0.945; p=0.019). Given concerns about the methodology of this study, this analysis was not used in the lifetime risk assessment.

*POT1* constitutional variants, and in particular missense variants that are located within functional domains and are considered damaging by functional predictors, have been identified in individuals with angiosarcoma, mainly cardiac (CAS) and breast. *POT1* variants were identified in 6/22 (27.3%) probands of *TP53*-negative LFL families with CAS. LFL families without CAS did not have *POT1* variants (0/34).^6^ While it is clear that individuals from families fulfilling clinical criteria of LFL who carry a *POT1* variant have an increased risk of developing AS, the penetrance of AS in POT1-TPDS outside an LFL family history has not yet been elucidated. More recently, multigene panel testing in a large cohort of patients demonstrated a statistically significant increased risk for all types of sarcomas (OR=6.6, 95% CI 3.1 to 13.9; p<0.001),^20^ but as above, there is caution about the methodology of this study and further work to clarify risk is likely required.

CLL has one of the strongest familial risks of haematological malignancies. Telomere dysregulation has been described as a key process in CLL development and pathogenic variants in *POT1* have been identified in familial CLL. In 2013, Ramsay *et al* reported that 3.5% (12/341) of CLL cases had somatic variants in *POT1.*^5^ However, Shi *et al* did not identify constitutional *POT1* variants in 70 familial CLL cases that underwent exome sequencing.^3^ Data from Speedy *et al* subsequently reported constitutional variants in *POT1* in 6% (4/66) of CLL families (at least two affected individuals).^21^ An additional case–control analysis by the same group showed that a missense *POT1* variant (NM_015450.3:c.1127A>G) conferred a 3.6-fold increase in the risk of CLL. Interestingly, this variant was recently reclassified as a variant of uncertain significance.^12^ Patients with POT1-TPDS develop CLL at a younger age compared with patients with incidental CLL (59 years vs 70 years). CLL families with *POT1* variants do not segregate with glioma or melanoma, suggesting that the penetrance of constitutional variants in *POT1* or other genes involved in the shelterin complex is modest and act as moderate penetrance alleles. A small number of case studies have recently been published linking POT1-TPDS with Hodgkin’s lymphoma,^22 23^ childhood acute myeloid leukaemia,^24^ splenic marginal zone lymphoma,^25^ multiple myeloma^26^ and myeloproliferative neoplasms.^27^

Data reporting an association of constitutional variants in *POT1* with familial glioma are limited. Constitutional variants in *POT1* were identified in two families with more than one individual with glioma (1% (3/301)). One or more affected family members had oligodendroglioma.^28^ A recent study by DeBoy *et al* reported two further glioma cases.^2^ There are no other case–control data in the literature. Colorectal and differentiated thyroid cancer have also been proposed as part of POT1-TPDS. However, the data around the prevalence of mutations are limited in these cancers. Chubb *et al* reported 3/1004 familial colorectal cancer cases.^29^ There are a total of 10 reported cases affected with thyroid cancer.^2 30 31^

### Recommendations

Following discussion at the expert group meetings, we propose a POT1-TPDS UK surveillance protocol (table 2) to be adopted by UK Clinical Genetics Services. A patient information leaflet (PIL) for individuals with *POT1* GPVs and their relatives has also been developed.^32^ The PIL raises patient awareness of the possibility of developing certain types of cancers and explains to patients their lifetime cancer risks compared with the general population. It can also empower patients to discuss likely associated health issues with their General Practitioner and other healthcare professionals. The rationale for the recommendations for each tumour type is detailed below.

**Table 2:** Surveillance recommendations for individuals with *POT1* GPVs

Surveillance recommendations for individuals with *POT1* **GPVs**		Evidence*
Cutaneous melanoma	Baseline full skin examination at diagnosis by a dermatologist for all individuals with *POT1* GPVs (age 18 or over).	Moderate
	Detailed discussion about symptom awareness and education on how to self-examine.	
	Contact information to be provided for reporting any new concerning symptoms and signs.	
	Decision on further surveillance to be made by a dermatologist based on individual’s phenotype and risk factor profile.†	
	Consideration of surveillance every 3–6 months for high-risk individuals tailored based on the individual and family.	
	Discharge from follow-up when appropriate and safe.	
Angiosarcoma/CAS	Not routinely recommended.	Moderate
	Cardiac MRI every 6–12 months should only be considered if there is a strong family history of CAS (≥1 FDR/SDR affected with CAS) (age 18 or over).	
CLL	Not recommended.	Moderate
Glioma	Not currently recommended.	Moderate
Other	Provide information on relevant resources.	
	Discuss the importance of making positive lifestyle choices.	

* Surveillance data should prospectively be collected and audited to inform future practice.

† Example: family history, early onset of melanoma in family, personal history, prior melanoma count, more than 100 moles on high resolution mole mapping, detection of at least 5 atypical moles etc).

CAS, cardiac angiosarcoma; CLL, chronic lymphocytic leukaemia; FDR, first-degree relative; GPVs, germline pathogenic variants; POT1, protection of telomeres 1; SDR, second-degree relative.

#### Rationale for recommendations

##### Cutaneous melanoma

It is recognised that complete cutaneous examination by dermatologists increases the diagnosis of early malignant melanoma, and that self-examination increases the rate of diagnosis of early lesions.^33 34^ There are a number of genetic conditions that can predispose to cutaneous melanoma with established screening strategies. Specialised clinics for individuals with an inherited predisposition to melanoma exist in a limited number of hospitals around the country dependant on local resources and infrastructure. Dedicated specialist clinics can offer an extended surveillance programme for individuals with increased melanoma risk, such as those with *CDKN2A* GPVs (a major melanoma susceptibility gene) or with a personal history of multiple early onset melanomas (where a melanoma gene panel is negative). Such a surveillance programme may include clinical review every 6–12 months (increased to 3–6 monthly review for those who have had a recent melanoma, more than one melanoma or additional risk factors), full skin examination by a dermatologist, high resolution mole mapping, advice about symptom awareness and access to a hotline, if patients notice any skin changes.

Taking into account the low absolute risk for developing cutaneous melanoma and the wide age of onset for first primary cutaneous melanoma (15–80 years),^8^ the opinion of the expert group was that routine surveillance in the form of annual or more frequent dermatological review is not required in the absence of a prior history of melanoma or additional melanoma risk factors. The group felt that a single baseline skin examination at diagnosis by a dermatologist should be offered with emphasis on detailed discussion of ‘red flag’ symptoms and self-monitoring. As the risk of the development of cutaneous melanoma is multifactorial, the consensus group agreed that taking into consideration various risk factors (eg, prior melanoma, skin type and colour, hair colour, mole number and morphology, sun exposure, degree of sunburn as a child, personal and family history of cancer), as well as detailed phenotyping of individuals with *POT1* GPVs would be beneficial in guiding further surveillance decision-making within a dermatology clinic.

##### Angiosarcoma

During the expert meetings the utility of various surveillance tools for CAS was discussed. Pertinent to the discussion is the fact that WB-MRI is currently not recommended for LFL families in the UK and that while WB-MRI is recommended for *TP53* heterozygotes,^35^ there is currently not equitable resourcing or access to WB-MRI across the UK. It was agreed that WB-MRI has limited utility in the detection of CAS. Although echocardiogram is widely available across the country, it has limited utility in detecting early CAS. With movement artefact and limited resolution, the tumours would likely have to be reasonably large to be detected, limiting the benefit of screening.

Cardiac MRI was considered a better method for CAS detection but has not been formally assessed in this setting. It was considered that cardiac MRI at a frequency of 6–12 months would be a reasonable surveillance tool for early detection of CAS, although the availability of cardiac MRI is limited to specialist centres. The group discussed that CAS symptoms usually appear in advanced disease and advice on ‘red flag’ symptom awareness is unlikely to be beneficial to individuals with *POT1* variants in improving early detection. Early disease detection with imaging could make a difference to patient outcomes, if CAS can be surgically resected early. A study addressing cardiac MRI in POT1-TPDS would be difficult to conduct given the small numbers of *POT1* heterozygotes and the small number who would go on to develop CAS. Overall, agreement was reached that surveillance in the form of cardiac MRI would be unlikely to be justified in most *POT1* heterozygotes, due to the small number of individuals with *POT1* GPVs who develop CAS. However, the group felt that cardiac MRI surveillance may be considered in *POT1* heterozygotes (age 18 or over) with a strong family history of CAS in ≥1 first or second-degree relative, as these individuals are likely to be at higher risk. Early detection of CAS may make a difference to the disease outcome in this scenario, but it is recognised that this approach is based on expert opinion only.

Local clinical genetics or sarcoma centres should consider referral for cardiac MRI to a specialist cardiothoracic centre as most local radiologists or cardiologists that report cardiac MRI do not have much experience in these. It can be challenging to visualise small cardiac tumours on MRI even when the tumour is known. Therefore, it was agreed that cardiac MRI should be undertaken and reported at a specialist cardiothoracic centre with optimised protocols/scanners and specific expertise in cardiac tumours.

WB-MRI would also have limited utility in breast AS screening. The expert group considered a dedicated breast MRI a better method for breast AS detection. In the UK, breast MRIs are used in LFS and in patients with very high-risk breast cancer. As the association of breast AS in POT1-TPDS is not yet clear, it was agreed that surveillance in the form of breast MRI cannot be justified.

##### Chronic lymphocytic leukaemia

There is weak evidence regarding CLL risk in POT1*-*TPDS. Surveillance in the form of annual full blood count (FBC) and physical examination including lymph nodes was not considered by the group to be helpful due to limited evidence to suggest that early detection would change management options or offer a survival benefit. Haematologists do not currently suggest FBC surveillance to relatives of familial CLL cases. Moreover, FBC surveillance is currently not recommended in the management guidelines for other genes that predispose to haematological malignancy.^36^ Therefore, the recommendation is for no additional surveillance over that offered to the general population. Symptom awareness was also not felt to be helpful with respect to early detection of CLL.

##### Glioma

The expert group felt that surveillance of the brain in the form of annual brain MRI should not be routinely offered as the absolute risk of oligodendroglioma among individuals with *POT1* GPVs appears to be low. Moreover, brain MRI can incidentally detect benign disease that could cause unnecessary anxiety to asymptomatic individuals with *POT1* variants and their families. However, in the presence of a strong family history of brain tumours, brain MRI surveillance could be considered, as detection of low-grade glioma prior to transformation to glioblastoma multiforme can influence length of survival.^37^

## Discussion

Recent studies suggest that *POT1* GPV are associated with a broader spectrum of hereditary cancer than the previously described POT1-TPDS.^9^ Recent international publications suggest employing a surveillance protocol similar to that used in LFS, particularly where there are LFS-like features.^8^ However, these studies are based on small numbers of individuals or families affected and do not support an equivalent lifetime cancer risk.

During the meeting, it was recognised that due to the rarity of POT1-TPDS and LP/P *POT1* variants, there is currently a limited evidence base to support surveillance recommendations in terms of early detection and cancer mortality. Our recommendations are based on the overall estimates of cancer risk and the potential utility of surveillance detecting cancer at an early stage where treatment options and/or overall survival would be improved. It was noted that there is a significant lack of evidence both for the cancer types and cancer risks for individuals with *POT1-*TPDS.

Perspectives on surveillance can differ between countries due to different experiences and health insurance systems. Our surveillance recommendations reflect the UK perspectives on the impact of surveillance on daily life and the cost and burden on our healthcare system. It was, therefore, recommended that discussion with patients includes a discussion of the limited information available, the balance of offering too much or too little surveillance and that future surveillance recommendations may change as more data become available. Individuals should be educated regarding general signs and symptoms of cancer and advised to seek medical attention with any concerning findings.

It is recognised that these recommendations will need to be reviewed and updated as we learn more about the spectrum of cancers and cancer risk in POT1-TPDS. In addition, there are recent reports suggesting that specific *POT1* variants may be associated with a different condition, dyskeratosis congenita, an inherited bone marrow failure condition characterised by specific mucocutaneous features.^38^ Further work will be required to establish genotype-phenotype correlations to inform clinical guidance.

### Next steps

Future surveillance protocols based on the accumulation of long-term, prospective data of *POT1* heterozygotes undergoing surveillance are necessary to avoid excessive and potentially unjustified burden. The UK Cancer Genetics Group (UKCGG), in collaboration with the National Disease Registration Service, is creating a national registry of individuals with inherited cancer predisposition that will include *POT1* heterozygotes. This will have the ability to link with other national registers, for example, cancer registry, to study the age and type of cancers occurring in individuals with *POT1* LP/P variants and potentially the clinical utility of the proposed surveillance recommendations. The sarcoma specialist meeting attendees would be interested in taking forward a more specific registry of unaffected individuals with *POT1* LP/P variants who are having cardiac MRI due to a strong family history of CAS (table 3). To ensure ongoing optimisation, we will re-evaluate the proposed surveillance recommendations as new information becomes available.

**Table 3:** Identified areas for further work

Ongoing discussion with the National Disease Registration Service (NDRS) to create a national registry of individuals with *POT1* GPVs.	UKCGG/NDRS
Create a national registry of individuals with *POT1* GPVs (unaffected, but with a family history of CAS) who are having cardiac MRI.	Sarcoma specialists attendees
Include individuals with *POT1* GPVs affected with melanoma in national registry and review long-term outcomes.	Dermatology specialists attendees

CAS, cardiac angiosarcoma; GPVs, germline pathogenic variants; POT1, protection of telomeres 1.

## Supplementary material

10.1136/jmg-2025-110638 online supplemental file 1
