# Correction: *UK clinical practice guidelines for the management of patients with constitutional *POT1* pathogenic variants*

**DOI:** 10.1136/jmg-2025-110638corr1

**Published:** 2026-07-16

Tsoulaki O, Evans DG, Sinha K, *et al.* UK clinical practice guidelines for the management of patients with constitutional *POT1* pathogenic variants. *Journal of Medical Genetics* 2025;62:559-565.

The name of author Farrah S Bakr has been corrected. Both the online PDF and HTML versions have been amended.

