# Supplementary material for: UK clinical practice guidelines for the management of patients with constitutional POT1 pathogenic variants
Source: J Med Genet. 2025 May 11;62(9):e110638. doi: 10.1136/jmg-2025-110638 (PMC12418547; doi:10.1136/jmg-2025-110638)
Supplement: online supplemental file 1 [file jmg-62-9-s001.docx]

**Table S1: Key points for discussion by the UK expert group regarding surveillance in individuals with constitutional *POT1* pathogenic/likely pathogenic variants.**

|  | **Points for discussion: UK expert group** | **Recommendations by Henry *et al* (last revision March 10, 2022) (3)** |
| --- | --- | --- |
| **Cutaneous melanoma** | **Lifetime risk**  ~3.3-5% for cutaneous melanoma  **Surveillance**   1. What is the utility of screening in the form of **annual dermatological review including full body examination and photography** **every 6 months** for *POT1* heterozygotes? 2. If there is utility, should the above screening be offered to all *POT1* heterozygotes or only to those who have additional risk factors (e.g. multiple atypical nevi etc)? 3. When should annual dermatology review start? At point of diagnosis or at age 18 years, depending on which is the latest? 4. If there is no utility of the above screening, should any additional screening be considered depending on the family history and/or judged on a case-by-case basis. 5. Should all *POT1* heterozygotes be offered **advice about symptom awareness** (e.g. self-check for moles). If yes, at what frequency? | **At diagnosis**  Full skin exam by dermatologist - beginning at age 18 years  **Further surveillance**  Dermatologic exam:   - Beginning at age 18 years at least every 6 months w/excision of any lesions suspicious for melanoma - Consider every 3 months in persons w/multiple atypical nevi, history of melanoma, &/or family history of melanoma |
| **Angiosarcoma** | **Lifetime risk**  ~2% for angiosarcoma  Case and family reports derive mainly from *TP53*-negative Li-Fraumeni-like (LFL) families with cardiac angiosarcoma  **Surveillance**  Note that Whole-body MRI (WB-MRI) is currently not recommended for LFL families in the UK.     1. What is the utility of screening in the form of either **WB-MRI or ECHO** for *POT1* heterozygotes? 2. If no utility of the above screening, should any additional screening be considered depending on the family history and/or judged on a case-by-case basis. 3. Should all *POT1* heterozygotes be offered **advice about symptom awareness?** | **At diagnosis**  Consider whole-body MRI - beginning at age 18 years:   - In persons in families fulfilling LFS or LFL criteria - In persons w/personal & family history of non-cutaneous, non-brain malignancies   **Further surveillance**  Whole-body MRI:   - Annually in families fulfilling LFS or LFL criteria beginning at age 18 years - Consider every 1-2 years depending on personal & family history of non-cutaneous, non-brain malignancies |
| **CLL** | **Lifetime risk**  ~1-2% for CLL  **Surveillance**   1. What is the utility of screening in the form of a **Full Blood Count (FBC) every 12 months** for *POT1* heterozygotes? 2. What is the utility of screening in the form of a **physical exam including lymph nodes every 12 months** for *POT1* heterozygotes? 3. If no utility in the above screening, should any additional screening be considered depending on family history and/or judged on a case-by-case basis? 4. Should all *POT1* heterozygotes be offered **advice about symptom awareness** (signs and symptoms of CLL)? | **At diagnosis**  CBC w/differential - beginning at age 18 yrs  Comprehensive physical exam including lymph nodes  In persons who undergo MRI for angiosarcoma screening or another reason: review of whole-body MRI for enlarged lymph nodes  **Further surveillance**  CBC w/differential - annually beginning at age 18 years  Comprehensive physical exam including lymph nodes - annually  Evaluate results of whole-body MRI for enlarged lymph nodes - when imaging is performed (e.g., in families fulfilling LFL criteria) |
| **Brain (glioma)** | **Lifetime risk**  Not known  No good case control data are available    **Surveillance**   1. What is the utility of screening in the form of a **brain MRI every 1-2 years** for *POT1* heterozygotes? 2. If no utility of the above screening, should any additional screening be considered depending on family history and/or judged on a case-by-case basis? 3. Should all *POT1* heterozygotes be offered **advice about symptom awareness**? | **At diagnosis**  Brain MRI w/& w/o contrast  Beginning at age 18 years  **Further surveillance**  Brain MRI: consider every 1-2 years depending on family history beginning at age 18 years |
